# Heavy Metal Characteristics and Their Association With Microbial Communities of Wetland Soils of Shengjin and Caizi Lakes, China

**DOI:** 10.1002/mbo3.70426

**Published:** 2026-09-30

**Authors:** Huiwu Geng, Gang Liu

**Affiliations:** ^1^ College of Life Sciences Anhui Medical University Hefei China

**Keywords:** Caizi Lake, diversity, heavy metals, microbial community, Shengjin Lake, soil

## Abstract

Wetland soils are among the ecosystems most vulnerable to anthropogenic disturbances, and they play critical roles in biogeochemical cycling and energy exchange. In this study, we investigated the characteristics of heavy metal distribution and microbial community structures in soil samples from Shengjin Lake and Caizi Lake in China. Analysis of soil physicochemical properties revealed a significant difference in soil pH between the two lakes, whereas no significant differences were detected in organic matter content or cation exchange capacity. Soils from Caizi Lake exhibited significantly higher concentrations of Pb, As, and Hg than those from Shengjin Lake, whereas Cd and Cr concentrations did not differ significantly between the two lakes. Soil microbial analysis demonstrated that Proteobacteria, Actinobacteriota, and Chloroflexi were the dominant phyla across all samples. Alpha diversity and principal coordinate analysis revealed significant differences in soil microbial communities between Shengjin and Caizi Lakes. LEfSe analysis indicated that *Pedobacter*, *Nocardioides*, and *Flavobacterium* were significantly enriched in the soils from Shengjin Lake, whereas *Arthrobacter* was significantly more abundant in the soils from Caizi Lake. To further explore the relationships between heavy metals and microbial communities, we analyzed the correlation heatmaps of the associations among heavy metals, microbial taxa, and KEGG functional pathways in samples from both lakes. Collectively, this study enhances the current understanding of heavy metal distribution and microbial community characteristics in wetland soils and provides a scientific basis for early environmental pollution warnings in the wetland ecosystems of Shengjin and Caizi Lakes.

## Graphical abstract

Heatmaps showing correlations between heavy metals and microbiota, heavy metals and KEGG pathways, and microbiota and KEGG pathways in Shengjin Lake and Caizi Lake, respectively.

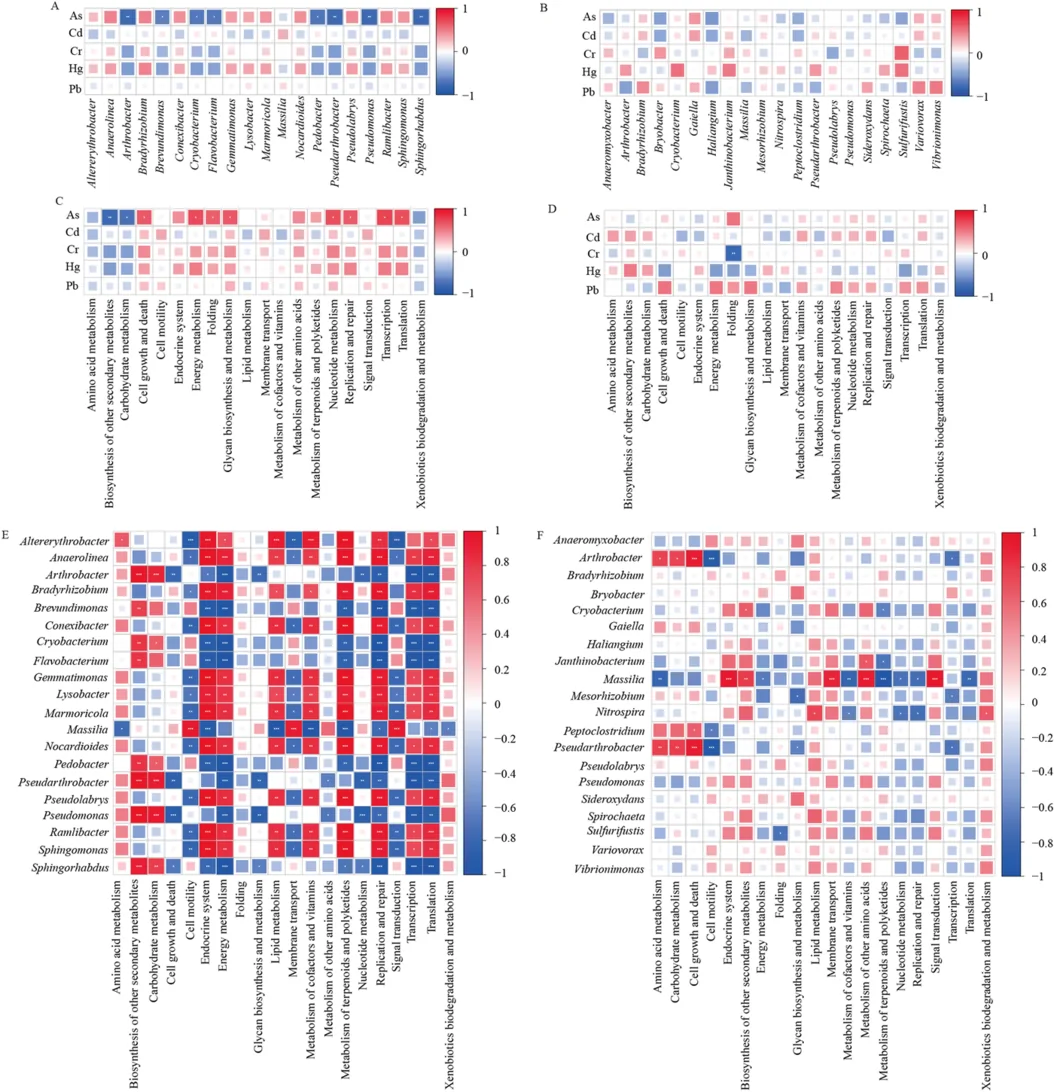

## 1 Introduction

Heavy metals are among the most critical pollutants in wetland ecosystems. Owing to their nonbiodegradable nature, high ecotoxicity, and strong environmental persistence, they are considered extremely hazardous contaminants in various ecosystems (Proshad et al. 2022). Consequently, global concern over the ecological risks posed by the heavy metal contamination of wetlands has been steadily increasing (Proshad et al. 2022; Wang et al. 2022). Heavy metal pollution in wetland soils is largely attributable to the unique susceptibility of wetlands to metal accumulation, transformation, and migration, which is closely associated with their inherent hydrological and geochemical properties (Yin et al. 2020; Xia et al. 2025). This vulnerability is primarily driven by fluctuating redox conditions and dynamic hydrological regimes, wherein alternating anoxic (flooded) and oxic (drained) conditions regulate the input, transport, speciation, toxicity, and accumulation of heavy metals, thereby shaping their subsequent ecological impacts on wetland organisms and ecosystem functions (Yin et al. 2020; Xia et al. 2025). Heavy metals in contaminated wetlands not only pose long‐term threats to soil organisms and ecosystem health but also increase bioaccumulation potential along food chains and webs, leading to significant toxicological risks for wildlife and humans (Chibuike et al. 2021; Huang et al. 2021; Schwantes et al. 2021; Hao et al. 2022).

Microbial communities are key components and biological indicators of wetland soil ecosystems that participate in diverse soil biochemical processes and play crucial roles in maintaining ecosystem stability (Khan et al. 2010). The composition and structure of soil microbial communities reflect wetland stability and support functional integrity, and a stable community is fundamental to maintaining wetland soil fertility (Zhou et al. 2015; Shi and Ma 2017; Huang et al. 2021). Microbial communities are essential for the transformation and recycling of chemical elements, and they also play critical roles in nitrogen fixation and plant cellulose degradation (Zhang et al. 2021; Wang et al. 2022). Owing to their high sensitivity to heavy metals, the structure and function of wetland soil microbial communities can shift under metal stress, as demonstrated by numerous studies on community composition and metabolic activities (Chen et al. 2018; Pacwa‐Płociniczak et al. 2018; Xiao et al. 2020; Zhu et al. 2023). Heavy metals typically impair soil microbial communities, leading to altered structure and reduced diversity. However, some metal‐tolerant microbial species can survive and restructure their communities, which is accompanied by an increase in their relative abundance (Xiao et al. 2020; Zhu et al. 2023). Soil microbes enzymatically convert toxic heavy metals into stable and less bioavailable forms (Borch et al. 2010; Gadd 2010). Furthermore, some soil bacteria facilitate metal precipitation and reductive immobilization, thereby alleviating heavy metal toxicity and maintaining the stability of wetland ecosystems (Maqsood et al. 2022; Meziane et al. 2026).

The characteristics of lake wetland soil ecosystems are closely associated with management practices and anthropogenic activities, which result in significant differences in heavy metal pollution profiles and microbial community structures across diverse lake wetlands (Tong et al. 2005). In recent decades, alongside rapid economic development, growing research attention has been devoted to heavy metal contamination and the associated shifts in microbial communities within wetland soils worldwide. However, critical knowledge is scarce regarding the distribution characteristics of heavy metals and the composition, diversity, and functional traits of soil microbial communities in the wetland soil ecosystems of Shengjin and Caizi Lakes in China. Accordingly, a systematic investigation of heavy metal accumulation patterns and microbial community features in the soils of these two lakes is of substantial scientific and practical significance for regional wetland protection and ecological risk assessment.

In the present study, 20 soil samples were collected from Shengjin Lake and Caizi Lake for integrated analysis. We proposed two core hypotheses: (1) heavy metal distribution characteristics and soil microbial community diversity differ significantly between Shengjin Lake and Caizi Lake, and (2) heavy metal pollution exerts measurable effects on the composition and structural assembly of soil microbial communities in the wetlands of both lakes. In this study, we aimed to conduct a comprehensive ecological risk assessment of soils from these two lakes, elucidate the response mechanisms of soil microbial communities under heavy metal stress, and offer novel insights into the distribution and adaptive patterns of the soil microbiota in distinct freshwater lakes.

## 2 Materials and Methods

### 2.1 Study Area, Sampling Locations, and Soil Sample Collection

Shengjin Lake (30.25°–30.50°N, 116.92°–117.25°E) and Caizi Lake (30.75°–30.97°N, 117.00°–117.15°E) are typical shallow lakes connected to the Yangtze River in Anhui Province, China. Shengjin Lake is located on the southern bank of the Yangtze River, whereas Caizi Lake is located on the left bank (Yang et al. 2015). The total area of Shengjin Lake is 33,300 hm^2^, whereas that of Caizi Lake is 16,667 hm^2^. The annual average temperature of Shengjin Lake ranges from 16.5°C to 16.7°C, and that of Caizi Lake is 16.5°C. The annual precipitation in Shengjin Lake is 1291.33–1322.23 mm, compared with 1200–1,389 mm in Caizi Lake (Wang et al. 2021). These two lakes are situated in a humid subtropical monsoon climate zone and function as crucial stopovers and wintering habitats for migratory waterbirds along the East Asian–Australian Flyway (Fox et al. 2011; Liu et al. 2024).

As typical shallow river‐connected lakes in the middle and lower reaches of the Yangtze River, Shengjin and Caizi Lakes have hydrological regimes closely linked to the water level of the Yangtze River. During the wet summer season, the lake water depth can reach 2.5–3 m. With the arrival of late autumn and early winter, the lake water level decreases in tandem with that of the Yangtze River. During the dry winter season, the water depth is approximately 30 cm, exposing large areas of lake beaches.

To obtain a comprehensive understanding of the characteristics of heavy metals and microbial communities in the wetland ecosystems of Shengjin Lake and Caizi Lake, a total of 20 soil samples were collected in December 2022 from both lakes, with 10 samples obtained from each lake. All sampling sites were required to be representative of the study area and were deliberately chosen to mainly focus on the exposed lakeshore grasslands where birds inhabit and forage; they were also as far away from human activities as possible (Figure 1, Supporting Information: Table S1). The sampling sites were mainly grassy mudflats, accounting for 68.01% of the total area. Grassy mudflats were dominated by *Carex* spp., with a total vegetation coverage of over 85%.

**Figure 1:**
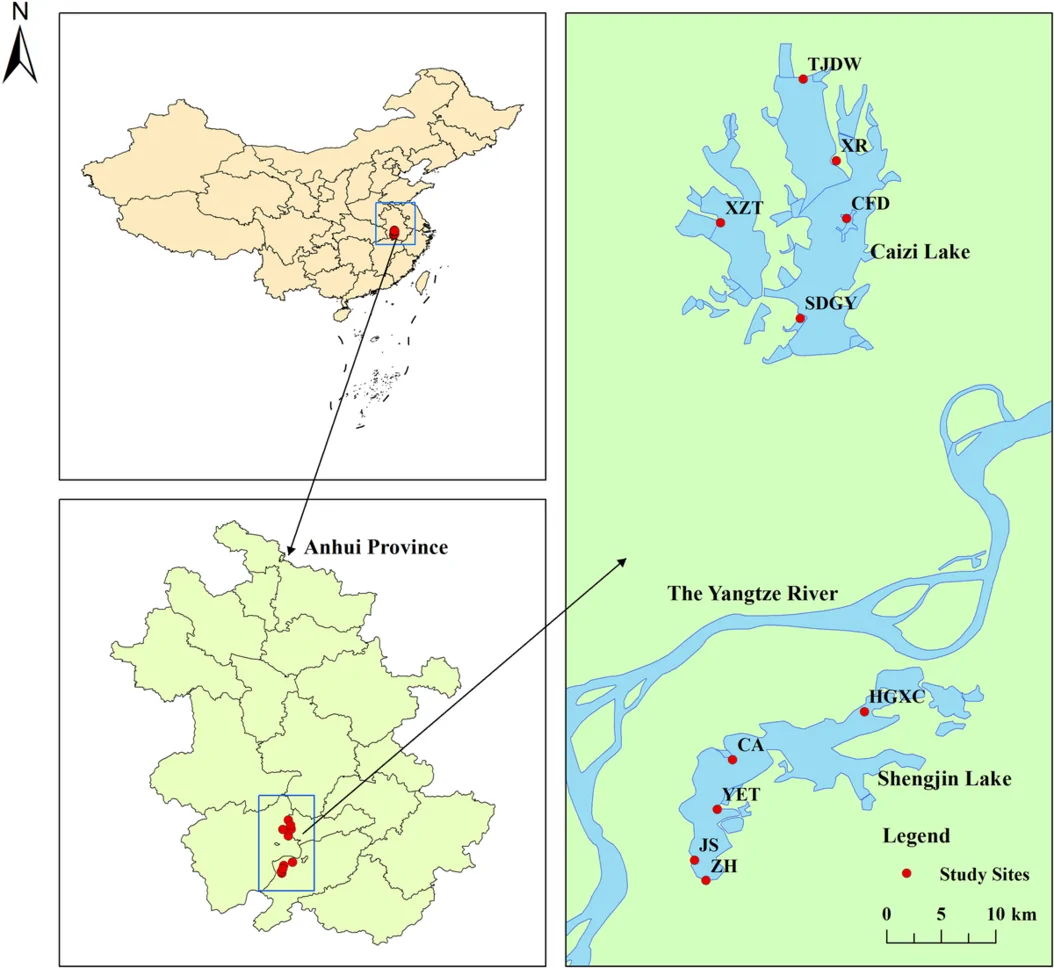
Soil sampling sites in Shengjin Lake and Caizi Lake. Site codes are shown in Supporting Information: Table S1.

At each sampling site, surface soil subsamples (0–20 cm depth) were collected from four corner points and one central point in a 20 × 20 m quadrat. Subsamples from these five points were thoroughly mixed and pooled into a single composite soil sample to ensure representativeness. All soil samples were immediately placed in sterile polyethylene bags, stored in insulated ice boxes to maintain a low temperature, and promptly transported to the laboratory, where they were stored at −80°C for subsequent analysis.

### 2.2 Determination of Chemical and Physical Properties of Soils

Plant tissue, stones, and other impurities in the soil samples were carefully discarded. The moist soil was spread in a clean sample tray, dried naturally in a ventilated room, and turned over twice daily. The soils were ground and passed through 2, 0.25, and 0.15 mm sieves sequentially to collect fine soils. The following data were collected from the soil samples that had been processed according to the requirements of the respective analyses. For soil pH determination, samples were mixed with decarbonated deionized water at a ratio of 1:2.5 (w/v), vortexed, and allowed to stand for 30 min. Soil pH was measured potentiometrically at 25°C using a glass electrode pH meter (FE28‐Standard; Mettler‐Toledo, Columbus, OH, USA) calibrated with pH 6.86 and pH 4.00 reference solutions (Mettler‐Toledo, Shanghai, China) before the test. The cation exchange capacity (CEC), which represents the total amount of exchangeable cations, was determined using the NH_4_Ac extraction method because of the acidic and neutral pH values of the soil samples. The procedure included multiple cycles of 1 M NH_4_AC extraction, centrifugation, and 95% (v/v) ethanol washing, followed by distillation and titration of the distilled products with a standard hydrochloric acid solution. Blank controls were used as quality controls. The CEC value was calculated using the formulation at the same volume as a hydrochloric acid solution. The soil organic carbon (SOC) content was quantified by sulphochromic oxidation. Each soil sample or an equal amount of silicon dioxide powder, which was used as a blank control, was sequentially mixed with potassium dichromate and sulfuric acid in a hard, heat‐resistant tube. The suspension was boiled in an oil bath for 5 min and titrated with ferrous sulfate. The SOC value was calculated using the formulation at the same volume as ferrous sulfate. Reagents of analytical grade or higher were used in these procedures.

### 2.3 Determination of Soil Heavy Metals

Microwave dissolution and atomic fluorescence spectrometry were used to determine As and Hg contents. The procedure was primarily as follows. Soil sample (0.25 g), 6 mL concentrated hydrochloric acid (HCl), and 2 mL concentrated nitric acid (HNO_3_) were added to a sample dissolution tank in sequence. The tank was shaken thoroughly to ensure uniform mixing, and the reaction was terminated if a severe reaction occurred. Next, the container was placed in a microwave dissolution instrument (M6; Preekem, Shanghai, China). The dissolution program was as follows: 120°C for 3 min, 160°C for 3 min, and 180°C for 25 min. After cooling to room temperature, the suspension and sediment were collected, filtered, and adjusted to predetermined volumes. The sample solution was diluted to an appropriate concentration and subjected to atomic fluorescence spectrometry (AFS‐230E; Haiguang, Beijing, China) in the following order: As or Hg standard solution (National Institute of Metrology, Beijing, China), which was used to plot a standard curve; reagent blank control; sample solution; and sample blank control.

The determination of Pb, Cd, and Cr was conducted using the HNO_3_‐HClO_4_‐HF triacid digestion method, with specific concentrations and detailed procedures as follows: 5 mL of concentrated nitric acid (HNO_3_, 65% v/v), 2 mL of perchloric acid (HClO_4_, 70% v/v), and 1 mL of hydrofluoric acid (HF, 40% v/v) were sequentially added to 0.5 g of the dried soil sample or silicon dioxide powder placed in a digestion tank. The mixture was first soaked at room temperature for 12 h to ensure complete infiltration of the reagents into the sample. The samples were then digested as previously described. The digestion solution was transferred to a 50 mL volumetric flask, and ultrapure water was added to dilute it to the specified volume, followed by thorough shaking to obtain the sample solution for Pb, Cd, and Cr determination. Before the analysis, the Ge internal standard (National Institute of Metrology, Beijing, China) was added to the standard and sample solutions. The standard solution (National Institute of Metrology, Beijing, China) was analyzed using a NexION 300X inductively coupled plasma mass spectrometer (ICP‐MS; Perkin Elmer, Shelton, CT, USA), and a standard curve was plotted based on the detection results. The instrument was rinsed with 1% nitric acid solution before the sample was loaded. Finally, the samples and blank controls were analyzed using the instrument. To verify the accuracy of the measurements, we tested a certified reference material for the chemical composition of soil (Reference No. GBW07453, Institute of Geophysical and Geochemical Exploration, Chinese Academy of Geological Sciences, Tianjin, China) during the sample determination process. All standard substances were certified (Supporting Information: Table S2), and all other reagents were of analytical grade or higher.

### 2.4 Evaluation of the Geoaccumulation Index

In this study, the geoaccumulation index (*Igeo*) was used to assess heavy metal pollution levels and potential ecological risks in the surface soils from Shengjin and Caizi Lakes. The background values for heavy metals in river sediments of the lower Yangtze River system were adopted as the reference baseline. The background concentrations were as follows: As, 9.4 mg·kg^−1^; Cd, 0.1 mg·kg^−1^; Cr, 69.4 mg·kg^−1^; Pb, 25.9 mg·kg^−1^; and Hg, 0.04 mg·kg^−1^ (Chen et al. 2012; Shen and Guo 2025). *Igeo* is calculated as log2(Cn/1.5Bn), where Cn represents the measured content of elements in the sample and Bn represents the corresponding geochemical background value.

### 2.5 Microbial Community Analysis

Microbial genomic DNA was extracted using the E.Z.N.A.® Soil DNA Kit (Omega Bio‐tek, Norcross, GA, USA) according to the manufacturer's protocol. The V3–V4 region of the bacterial 16S rRNA gene was amplified by polymerase chain reaction (PCR) using the primer set 341 F (5′‐CCT AYG GGR BGC ASC AG‐3′) and 806 R (5′‐GGA CTA CNN GGG TAT CTA AT‐3′). The PCR thermal cycling conditions were as follows: initial denaturation at 95°C for 2 min; 25 cycles of denaturation at 95°C for 30 s, annealing at 55°C for 30 s, and extension at 72°C for 30 s; and a final extension step at 72°C for 5 min.

PCRs were performed on the ABI GeneAmp® 9700 system (ABI, Carlsbad, CA, USA) in triplicate in 20 μL mixtures containing 4 μL of 5× FastPfu Buffer, 2 μL of 2.5 mM dNTPs, 0.8 μL of each primer (5 μM), 0.4 μL of FastPfu Polymerase (TransGen Biotech, Beijing, China), and 10 ng of template DNA. Amplicons were separated on 2% agarose gels and purified using an AxyPrep DNA Gel Extraction Kit (Axygen Biosciences, Union City, CA, USA) according to the manufacturer's protocol. Purified PCR products were pooled in equimolar concentrations, and an Illumina paired‐end sequencing library was constructed according to the standard genomic library preparation procedure. Finally, the amplicon library was subjected to paired‐end sequencing on a DNBSEQ‐G99 PE300 platform (MGI Tech, Shenzhen, China) following the manufacturer's standard protocols.

### 2.6 Bioinformatics, Association, and Statistical Analysis

Raw microbial sequencing data were obtained in the FASTQ format. Operational taxonomic units (OTUs) were clustered at a 97% similarity threshold using UPARSE (version 7.1), and chimeric sequences were identified and removed using UCHIME (Amato et al. 2013). Representative sequences of each OTU were selected using the QIIME2 platform. All the representative sequences were taxonomically annotated and aligned against the SILVA 16S rRNA reference database (Release 138.1; http://www.arb-silva.de). Rarefaction analysis was performed using Mothur v.1.21.1. The Chao1 diversity index was calculated using the “vegan” package in R v4.0.2, and principal coordinate analysis (PCoA) was conducted based on the Bray–Curtis distance matrix using the “ape” package in R v4.0.2 (Schloss et al. 2009; Lozupone et al. 2011). Linear discriminant analysis effect size (LEfSe) was used to identify potential biomarkers from the high‐dimensional microbial community data (Segata et al. 2011).

Heatmaps depicting the correlations between heavy metals and microbial taxa, heavy metals and Kyoto Encyclopedia of Genes and Genomes (KEGG) pathways, and microbial taxa and KEGG pathways were constructed on the Tutool platform (https://www.cloudtutu.com) based on Spearman's rank correlation, with significance set at *p* < 0.05. Between‐group comparisons were performed using GraphPad Prism v7.0 and SPSS 22.0. All data are presented as mean ± standard deviation (SD), and differences were considered statistically significant at *p* < 0.05.

## 3 Results

### 3.1 Soil Physicochemical Properties

Soil physicochemical properties, including pH, organic matter content, and CEC, were analyzed in soil samples from Shengjin and Caizi Lakes. Soil pH ranged from 5.98 to 6.90 in Shengjin Lake and from 5.44 to 6.13 in Caizi Lake, with a significant difference between the two lakes (Figure 2A). However, no significant differences between lakes were observed for organic matter content (Shengjin Lake, 11.55–33.83 g∙kg^−1^; Caizi Lake, 13.40–42.05 g∙kg^−1^; Figure 2B) or CEC (Shengjin Lake, 10.92–16.73 mol∙kg^−1^; Caizi Lake, 10.21–15.67 cmol∙kg^−1^; Figure 2C).

**Figure 2:**
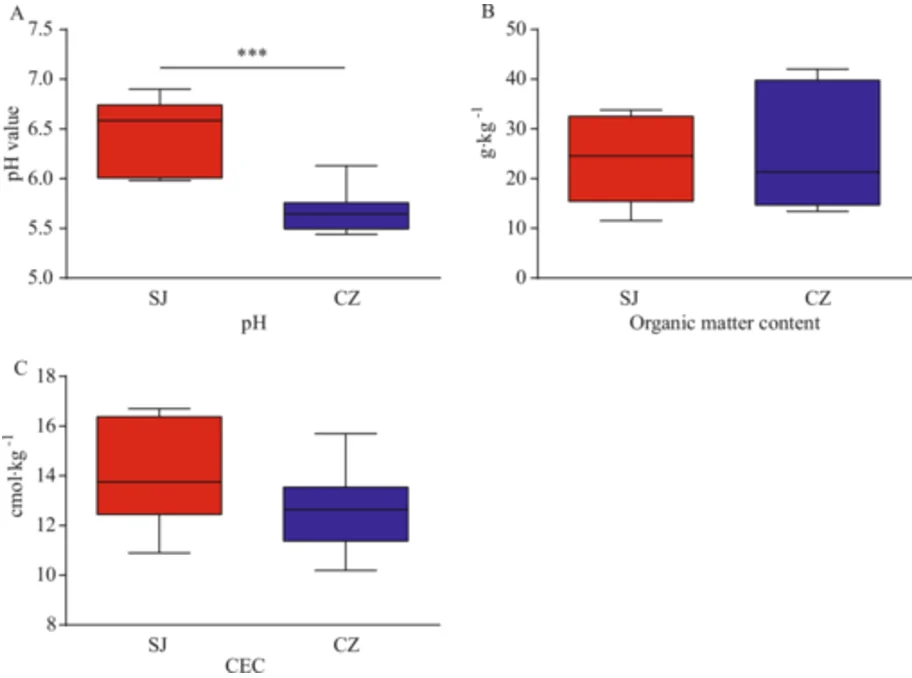
Soil physicochemical properties, including pH (A), organic matter content (B), and cation exchange capacity (CEC) (C) in soil samples from Shengjin Lake (SJ) and Caizi Lake (CZ).

### 3.2 Heavy Metal Concentrations

The average concentrations of the five heavy metals across all 20 soil samples from both lakes followed the order Cr > Pb > As > Cd > Hg. The mean concentrations of all five heavy metals were higher in soils from Caizi Lake than in those from Shengjin Lake (Table 1). Cr showed the highest concentrations, with mean values of 48.85 ± 16.99 mg∙kg^−1^ in Shengjin Lake and 66.64 ± 9.23 mg∙kg^−1^ in Caizi Lake. Hg exhibited the lowest concentrations, at 0.03 ± 0.004 mg∙kg^−1^ and 0.05 ± 0.004 mg∙kg^−1^ in Shengjin and Caizi Lakes, respectively. The concentrations of Pb, As, and Hg were significantly higher (*p* = 0.003, 0.015, and 0.00001, respectively) in Caizi Lake soils than in Shengjin Lake soils, whereas Cd and Cr concentrations did not differ significantly (*p* = 0.381 and 0.052, respectively) between the two lakes. All five heavy metals (As, Cd, Cr, Hg, and Pb) exhibited negative *Igeo* values, suggesting that there was no significant heavy metal contamination in either lake (Table 1). To ensure accuracy, a certified reference soil material was analyzed after the 20 samples, and the results are presented in Supporting Information: Table S3. By comparing the instrument readings with the certified values, the relative errors were consistently below 6%. The sample detection results were therefore well supported.

**Table 1:** Concentrations, *Igeo* values, and corresponding pollution degrees of five heavy metals in soils from Shengjin Lake and Caizi lake.

Heavy metal	Shengjin lake			Caizi lake		
	Concentration (mg·kg^−1^)	*Igeo*	Pollution degree	Concentration (mg·kg^−1^)	*Igeo*	Pollution degree
As	8.80 ± 0.99^a^	−0.68	Uncontaminated	10.39 ± 1.42^b^	−0.44	Uncontaminated
Cd	0.09 ± 0.01^a^	−0.74	Uncontaminated	0.10 ± 0.01^a^	−0.59	Uncontaminated
Cr	48.85 ± 16.99^a^	−1.09	Uncontaminated	66.64 ± 9.23^a^	−0.64	Uncontaminated
Hg	0.03 ± 0.004 ^A^	−1.00	Uncontaminated	0.05 ± 0.004^B^	−0.26	Uncontaminated
Pb	9.12 ± 3.55 ^A^	−2.09	Uncontaminated	16.20 ± 5.13^B^	−1.26	Uncontaminated

*Note:* (1) *Igeo* classification: *Igeo* < 0: Uncontaminated; 0 ≤ *Igeo* < 1: Uncontaminated to moderately contaminated; 1 ≤ *Igeo* < 2: Moderately contaminated; 2 ≤ *Igeo* < 3: Moderately to heavily contaminated; 3 ≤ *Igeo* < 4: Heavily contaminated; 4 ≤ *Igeo* < 5: Heavily to extremely contaminated; *Igeo* ≥ 5: Extremely contaminated.

(2) Different lowercase letters (a, b) indicate significant differences at *p* < 0.05; different uppercase letters (A, B) indicate significant differences at *p* < 0.01. Values followed by the same letter are not significantly different (*p* > 0.05).

### 3.3 Soil Microbial Community

After quality filtering, the clean reads for each sample exceeded 50,000, indicating that the sequencing depth was sufficient to characterize the microbial community composition. The rarefaction curves for all samples approached the saturation plateau (Figure 3A), demonstrating that the sequencing depth was adequate to obtain stable and representative estimates of species richness.

**Figure 3:**
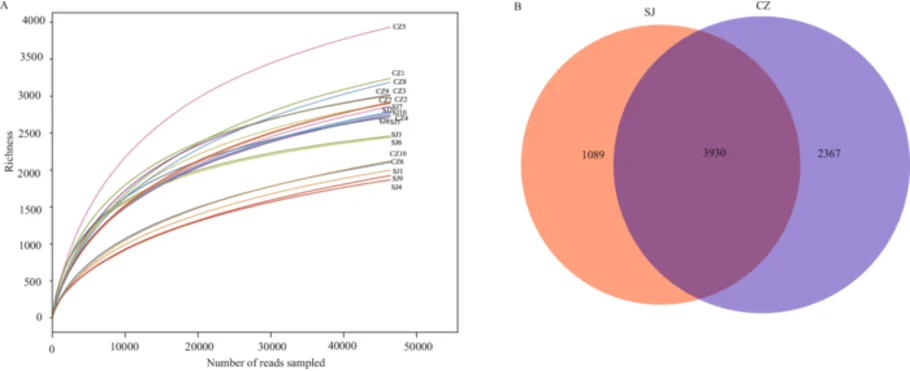
Rarefaction curves reflecting sequencing depth (A) and Venn diagram (B) showing the distribution of OTUs in soil microbial communities from Shengjin Lake (SJ) and Caizi Lake (CZ).

A total of 1,234,284 high‐quality reads were retained, corresponding to 11,316 OTUs. On average, 565.8 OTUs were identified per sample, and 3930 OTUs were shared between the two lakes. Shengjin Lake soil samples harbored 2367 unique OTUs, whereas Caizi Lake soil samples contained 1089 unique OTUs (Figure 3B).

Proteobacteria, Actinobacteriota, and Chloroflexi were the dominant bacterial phyla, accounting for 31.7%, 28.4%, and 10.9% of the total community, respectively (Supporting Information: Figure 1A). At the class level, Gammaproteobacteria (12.7%), Actinobacteria (12.5%), and Anaerolineae (5.6%) were the most abundant phyla (Supporting Information: Figure 1B). The top 10 bacterial orders were Burkholderiales, Micrococcales, Saccharimonadales, Anaerolineales, Rhizobiales, Sphingobacteriales, Propionibacteriales, Sphingomonadales, Flavobacteriales, and Gemmatimonadales (Supporting Information: Figure 1C). The dominant families were Oxalobacteraceae, Micrococcaceae, Anaerolineaceae, Sphingobacteriaceae, Microbacteriaceae, Nocardioidaceae, Sphingomonadaceae, Flavobacteriaceae, Xanthobacteraceae, and Gemmatimonadaceae (Supporting Information: Figure 1D). The 10 most abundant taxa at the genus level were *Massilia*, *Arthrobacter*, *Pseudarthrobacter*, *Pedobacter*, *Cryobacterium*, *Flavobacterium*, *Nocardioides*, *Anaerolinea*, *Gemmatimonas*, and *Pseudomonas* (Supporting Information: Figure 1E).

The Chao1 index was used to estimate the alpha diversity of soil microbial communities. Significant differences were observed between Shengjin Lake and Caizi Lake samples, with the microbial diversity being significantly higher in Caizi Lake soils (*p* = 0.029; Figure 4A). PCoA based on Bray–Curtis distances showed clear and distinct clustering of samples according to lake (PERMANOVA: *R* ^2^ = 0.26, *p* = 0.001; Figure 4B).

**Figure 4:**
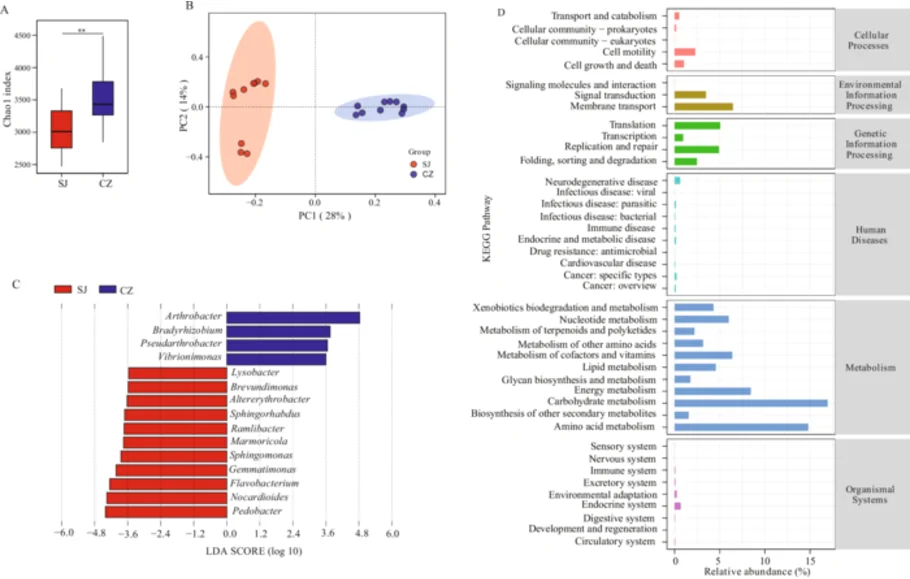
Comparisons of the Chao1 index (A), PCoA analysis (B), LEfSe analysis (C), and KEGG functional annotation (D) of soil microbial communities between Shengjin Lake (SJ) and Caizi Lake (CZ).

LEfSe analysis was performed to identify differentially abundant microbial taxa between the two lakes. *Pedobacter*, *Nocardioides*, and *Flavobacterium* were significantly enriched in Shengjin Lake soils, whereas *Arthrobacter* was significantly more abundant in Caizi Lake soils (Figure 4C).

Furthermore, PICRUSt‐based functional prediction revealed significant differences in microbial functions between the two lakes. A total of 42 KEGG orthologues were identified, covering pathways related to cellular processes, environmental information processing, genetic information processing, human diseases, metabolism, and organismal systems (Figure 4D).

### 3.4 Correlation Analysis

Heatmaps were generated to visualize pairwise correlations among heavy metals, microbial communities, and KEGG pathways across samples from Shengjin and Caizi Lakes. Spearman's rank correlation was used to evaluate the association between heavy metals and the top 20 most abundant soil microbial genera.

In Shengjin Lake soils, As was strongly negatively correlated with *Arthrobacter*, *Brevundimonas*, *Cryobacterium*, *Flavobacterium*, *Pseudarthrobacter*, *Pedobacter*, *Pseudomonas*, and *Sphingorhabdus* (Figure 5A). In Caizi Lake soils, no significant correlations were observed between the five heavy metals and the top 20 microbial genera (Figure 5B).

**Figure 5:**
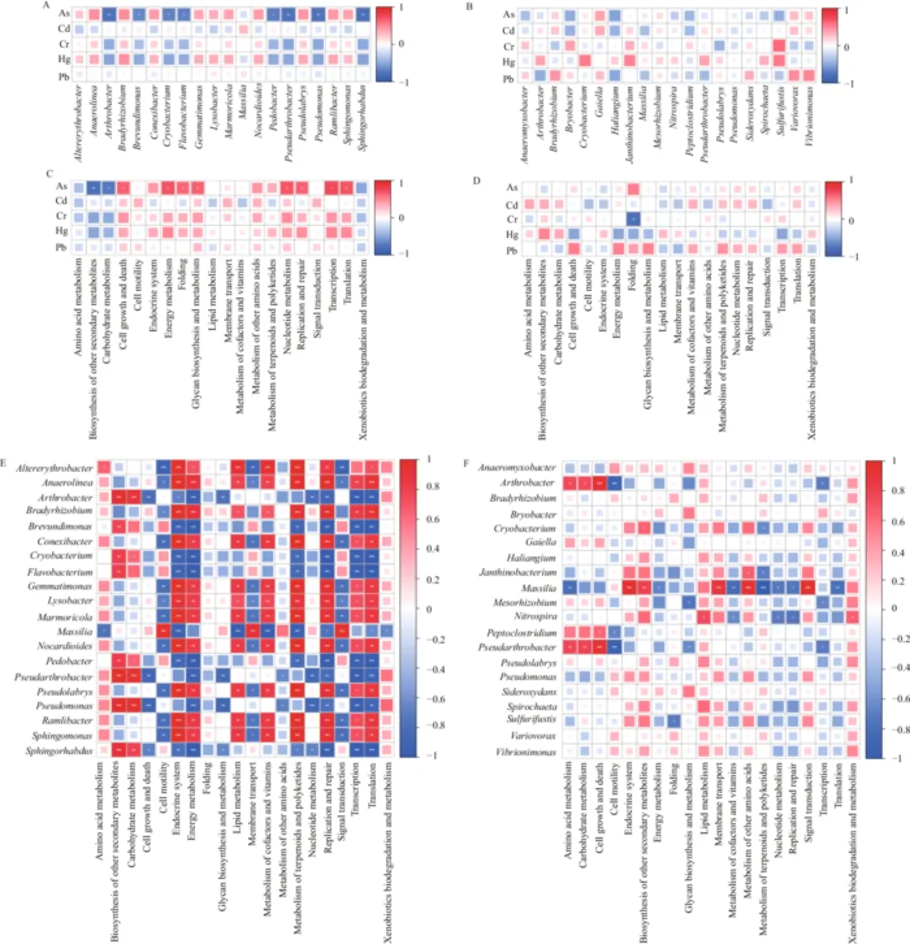
Heatmaps showing correlations between heavy metals and microbiota (A, B), heavy metals and KEGG pathways (C, D), and microbiota and KEGG pathways (E, F) in Shengjin Lake (A, C, E), and Caizi Lake (B, D, F), respectively.

The correlations between heavy metals and the top 20 KEGG functional pathways were also analyzed. In Shengjin Lake, As exhibited strong negative correlations with carbohydrate metabolism and the biosynthesis of other secondary metabolites, but strong positive correlations with cell growth and death, glycan biosynthesis and metabolism, protein folding, energy metabolism, replication and repair, nucleotide metabolism, translation, and transcription (Figure 5C). In Caizi Lake, Cr showed a strong negative correlation with protein folding (Figure 5D).

The correlations between the 20 most abundant microbial genera and the top 20 KEGG pathways were further examined. More than 20 significant positive and negative correlations between microbial genera and functional pathways were detected in the soils of both Shengjin Lake and Caizi Lakes (Figure 5E,F).

## 4 Discussion

In the present study, soil physicochemical properties and heavy metal concentrations were systematically investigated in Shengjin and Caizi Lakes. The results indicated that the heavy metal contents in the soils of both lakes were lower than those previously reported in the sediments of Chaohu, Poyang, and Taihu Lakes in the lower reaches of the Yangtze River (Yang et al. 2013). The soils were unpolluted and exhibited a low ecological risk level, demonstrating that the overall ecological safety of the two lakes was favorable. Compared to the sedimentary and heavy metal concentrations reported for the lower main stream of the Yangtze River, the levels of As, Cd, Cr, Hg, and Pb in both lakes were considerably lower than those found in the surrounding regions (Yang et al. 2013; Jin et al. 2024). Similarly, heavy metal concentrations in the lakeshore grassland soils in this study were much lower than those in the sediments of Shengjin Lake and Caizi Lake, as documented in previous studies (Jiang et al. 2018; Zhang et al. 2019).

Heavy metal accumulation in the middle and lower reaches of the Yangtze River is closely associated with anthropogenic activities, with major external sources, including surface runoff, industrial and agricultural emissions, and atmospheric deposition (Li et al. 2025). In this study, relatively higher concentrations of As, Hg, and Pb were observed in Caizi Lake. This difference may be attributed to the fact that Shengjin Lake is a well‐protected national nature reserve, whereas Caizi Lake faces more intensive human disturbances such as agricultural production, inland waterway transportation, and aquaculture activities. In addition, given the close proximity of farmland to both lakes, agricultural fertilizers, pesticides, and domestic wastewater‐derived pollutants (e.g., cleaning agents, toiletries, and cosmetics) are potential contributors to elevated levels of heavy metals (As, Cd, Cr, Hg, and Pb) in lakeshore soils (Alloway 2013). However, because this study did not include source apportionment analysis or sediment composition to distinguish anthropogenic inputs from naturally occurring geogenic metals, the pollution source attributions could not be definitively confirmed.

Soil microbial communities often exhibit distinct responses to heavy metal contamination, and variations in heavy metal concentrations can directly influence bacterial growth, reproduction, and community assembly (Giller et al. 2009; Sun et al. 2017). In this study, significant differences in soil microbial community structure were observed between Shengjin and Caizi Lakes. The Chao1 index revealed higher microbial species richness in Caizi Lake soils than in Shengjin Lake soils, suggesting that the richness and diversity of microbial communities in different wetland soils are not necessarily synchronized. The higher Chao1 index in Caizi Lake soils indicated a more species‐rich microbial community. Favorable soil pH and relatively high organic matter content may provide diverse microhabitats, reduce environmental stress, and support the survival and proliferation of a wide range of microbial taxa (Zhang et al. 2019). Such favorable habitat conditions further promote the enrichment of specific functional guilds. The synergistic interaction between soil physicochemical properties and microbial colonization ultimately contributes to the significantly higher species richness observed in Caizi Lake.

Microbial communities generally exhibit distinct responses to heavy metal stress in soil, and heavy metal concentrations can directly influence bacterial growth, reproduction, and community composition (Giller et al. 2009; Sun et al. 2017). Under heavy metal exposure, bacterial communities often develop a high number of unique OTUs because of adaptive selection (Sun et al. 2016, 2017; Li et al. 2021). In the present study, Proteobacteria and Actinobacteriota were identified as the dominant phyla in the soils from both lakes. Proteobacteria represent one of the most abundant and functionally diverse bacterial phyla in terrestrial ecosystems, participating extensively in carbon, nitrogen, and phosphorus cycling and playing essential roles in maintaining soil structure and regulating biogeochemical processes (Kim et al. 2021). Many members of Proteobacteria secrete extracellular polysaccharides and functional enzymes that enhance soil aggregation and accelerate organic matter decomposition, thereby sustaining soil health and ecological stability (Guan et al. 2023). In heavy metal–contaminated environments, Proteobacteria display strong adaptability and contribute significantly to metal detoxification and bioremediation. Previous studies have reported a significant positive correlation between the relative abundance of Proteobacteria and the remediation efficiency of heavy metal–polluted soils, highlighting their critical potential for ecological restoration (Li et al. 2023). Actinobacteriota also play a crucial role in the maintenance of soil ecosystem stability. They decompose refractory organic compounds such as cellulose and chitin, promote nutrient cycling, produce antibiotics to suppress soil‐borne pathogens, and facilitate humus formation and soil buffering capacity (Correa‐Garcia et al. 2023). Under heavy metal stress, Actinobacteriota exhibit high tolerance and bioremediation potential through biosorption, intracellular bioaccumulation, biotransformation, and bioleaching. These mechanisms include metal adsorption via cell wall functional groups, intracellular sequestration, and the conversion of toxic metal ions into less bioavailable forms, making certain taxa promising bioaugmentation agents for contaminated soils (Zuo et al. 2023; Kou et al. 2023).

As Shengjin Lake and Caizi Lake are internationally important wintering wetlands along the East Asian–Australasian Flyway, they support hundreds of thousands of waterbirds, including endangered species such as *Ciconia boyciana*, *Grus monacha*, and *Grus leucogeranus* (Liu et al. 2025). These waterbirds are highly vulnerable to heavy metal exposure within their habitats, which may lead to heavy metal bioaccumulation and subsequent sublethal physiological impairments. Heavy metals in wetland soils and their cascading effects on microbial communities are critically important for wetland ecological security and pose direct risks to migratory waterbirds (Liu et al. 2015; Xia et al. 2021). Therefore, characterizing the functional responses of soil microbes to heavy metal stress can help establish an early warning system for habitat degradation, thereby providing a scientific basis for the conservation of migratory bird populations and sustainable management of these ecologically vital wetlands.

In summary, the heavy metals detected in the soils of Shengjin and Caizi Lakes were primarily associated with agricultural and domestic pollution. The application of fertilizers and pesticides, along with the use of household products, may contribute to the release of heavy metals into the soils surrounding the two lakes, which is consistent with findings from other lakes located in agricultural areas. Our results demonstrate significant differences in the composition and diversity of soil microbial communities between Shengjin Lake and Caizi Lake, which may be driven by variations in heavy metal concentrations between the two lakes.

This study had several limitations. First, sampling was confined to the month of December, which provided only a snapshot of the microbial community responses across the study site. Second, microbial functional predictions were based on bioinformatic inference rather than direct experimental measurements (e.g., metagenomic or metatranscriptomic sequencing data), which limits the robustness of our conclusions regarding microbial functional responses to heavy metal stress. Third, the sample size was relatively small (20 soil samples from the 2 lakes), and spatiotemporal variability was not fully considered. These limitations should be addressed in future studies by conducting multi‐season sampling, incorporating additional physicochemical variables, performing direct functional profiling, and expanding the spatial and temporal coverage to enhance the generalizability of the findings.

## Author Contributions

**Huiwu Geng:** investigation, methodology, software, data curation, writing – original draft. **Gang Liu:** conceptualization, funding acquisition, writing – review and editing, writing – original draft.

## Ethics Statement

The authors have nothing to report.

## Conflicts of Interest

The authors declare no conflicts of interest.

## Supporting information


**Figure S1:** Dominant microbial taxa at the phylum (A), class (B), order (C), family (D), and genus (E) levels in soil samples from Shengjin Lake (SJ) and Caizi Lake (CZ). **Table S1:** Soil sampling sites and corresponding sample sizes collected from Shengjin Lake and Caizi Lake. **Table S2:** Standard solutions for plotting the curves. **Table S3:** Measurements of the required metal concentrations of the certified reference material.

## Data Availability

Data is deposited in the National Microbiology Data Center (NMDC, https://nmdc.cn/) with accession numbers NMDC40060970‐ NMDC40060989.

## References

[mbo370426-bib-0001] Alloway, B. J. , ed. 2013. Heavy Metals in Soils: Trace Metals and Metalloids in Soils and Their Bioavailability, 3rd ed. Springer Netherlands.

[mbo370426-bib-0002] Amato, K. R. , C. J. Yeoman , A. Kent , et al. 2013. “Habitat Degradation Impacts Black Howler Monkey (*Alouatta pigra*) Gastrointestinal Microbiomes.” ISME Journal 7: 1344–1353. 10.1038/ismej.2013.16 PMC3695285 23486247

[mbo370426-bib-0003] Borch, T. , R. Kretzschmar , A. Kappler , et al. 2010. “Biogeochemical Redox Processes and Their Impact on Contaminant Dynamics.” Environmental Science & Technology 44, no. 1: 15–23. 10.1021/es9026248 20000681

[mbo370426-bib-0004] Chen, X. , F. Chen , S. Jia , et al. 2012. “Soil Geochemical Baseline and Background in Yangtze River– Huaihe River Basin of Anhui Province.” Geology in China 39, no. 2: 302–310 (in Chinese with English Abstract).

[mbo370426-bib-0005] Chen, Y. , Y. Jiang , H. Huang , et al. 2018. “Long‐Term and High‐Concentration Heavy‐Metal Contamination Strongly Influences the Microbiome and Functional Genes in Yellow River Sediments.” Science of the Total Environment 637–638: 1400–1412. 10.1016/j.scitotenv.2018.05.109 29801233

[mbo370426-bib-0006] Chibuike, M. P. , N. D. Ogbonna , and W. J. Olufunmilayo . 2021. “Microbiology and Heavy Metal Content of Wetlands Impacted by Crude Oil Pollution in Rivers State, Southern Nigeria.” Microbiology Research Journal International 31: 53–63.

[mbo370426-bib-0007] Correa‐Garcia, S. , V. Corelli , J. Tremblay , et al. 2023. “Soil Fauna‐Microbial Interactions Shifts Fungal and Bacterial Communities Under a Contamination Disturbance.” PLoS One 18, no. 10: e0292227. 10.1371/journal.pone.0292227 PMC10599570 37878639

[mbo370426-bib-0008] Fox, A. D. , L. Cao , Y. Zhang , et al. 2011. “Declines in the Tuber‐Feeding Waterbird Guild at Shengjin Lake National Nature Reserve, China‐A Barometer of Submerged Macrophyte Collapse.” Aquatic Conservation: Marine and Freshwater Ecosystems 21: 82–91.

[mbo370426-bib-0009] Gadd, G. M. 2010. “Metals, Minerals and Microbes: Geomicrobiology and Bioremediation.” Microbiology 156, no. Pt 3: 609–643. 10.1099/mic.0.037143-0 20019082

[mbo370426-bib-0010] Giller, K. E. , E. Witter , and S. P. McGrath . 2009. “Heavy Metals and Soil Microbes.” Soil Biology and Biochemistry 41: 2031–2037.

[mbo370426-bib-0011] Guan, Y. , M. Wu , S. Che , et al. 2023. “Effects of Continuous Straw Returning on Soil Functional Microorganisms and Microbial Communities.” Journal of Microbiology 61, no. 1: 49–62. 10.1007/s12275-022-00004-6 36701105

[mbo370426-bib-0012] Hao, M. , Q. Zuo , J. Li , S. Shi , B. Li , and X. Zhao . 2022. “A Comprehensive Exploration on Distribution, Risk Assessment, and Source Quantification of Heavy Metals in the Multi‐Media Environment From Shaying River Basin, China.” Ecotoxicology and Environmental Safety 231: 113190. 10.1016/j.ecoenv.2022.113190 35032730

[mbo370426-bib-0013] Huang, X. , X. Wu , X. Tang , et al. 2021. “Distribution Characteristics and Risk of Heavy Metals and Microbial Community Composition Around the Wanshan Mercury Mine in Southwest China.” Ecotoxicology and Environmental Safety 227: 112897. 10.1016/j.ecoenv.2021.112897 34655882

[mbo370426-bib-0014] Jiang, Z. , N. Xu , B. Liu , et al. 2018. “Metal Concentrations and Risk Assessment in Water, Sediment and Economic Fish Species With Various Habitat Preferences and Trophic Guilds From Lake Caizi, Southeast China.” Ecotoxicology and Environmental Safety 157: 1–8. 10.1016/j.ecoenv.2018.03.078 29605640

[mbo370426-bib-0015] Jin, Y. , Y. Jiang , Q. Zhou , et al. 2024. “Characteristics and Ecological Risk Assessment of Sedimentary Heavy Metals From the Lower Mainstream of Yangtze River.” Geology in China 51, no. 1: 276–289 (in Chinese with English Abstract).

[mbo370426-bib-0016] Khan, S. , A. El‐Latif hesham , M. Qiao , S. Rehman , and J. Z. He . 2010. “Effects of Cd and Pb on Soil Microbial Community Structure and Activities.” Environmental Science and Pollution Research 17: 288–296. 10.1007/s11356-009-0134-4 19333640

[mbo370426-bib-0017] Kim, H. S. , S. H. Lee , H. Y. Jo , K. T. Finneran , and M. J. Kwon . 2021. “Diversity and Composition of Soil Acidobacteria and Proteobacteria Communities as a Bacterial Indicator of Past Land‐Use Change From Forest to Farmland.” Science of the Total Environment 797: 148944. 10.1016/j.scitotenv.2021.148944 34298360

[mbo370426-bib-0018] Kou, B. , Y. He , Y. Wang , et al. 2023. “The Relationships Between Heavy Metals and Bacterial Communities in a Coal Gangue Site.” Environmental Pollution 322: 121136. 10.1016/j.envpol.2023.121136 36736561

[mbo370426-bib-0019] Li, J. , M. Zhu , Y. Zhang , et al. 2025. “The Spatial Distribution and Risk Assessment of Nutrient Elements and Heavy Metal Pollution in Sediments: A Case Study of a Typical Urban Lake in the Middle and Lower Reaches of the Yangtze River.” Water 17, no. 14: 2094.

[mbo370426-bib-0020] Li, J. M. , J. H. Ou , F. Verpoort , R. Y. Surmpalli , W. Y. Huang , and C. M. Kao . 2023. “Toxicity Evaluation of a Heavy‐Metal‐Polluted River: Pollution Identification and Bacterial Community Assessment.” Water Environment Research 95, no. 7: e10904. 10.1002/wer.10904 37350694

[mbo370426-bib-0021] Li, Y. , M. Zhang , R. Xu , et al. 2021. “Arsenic and Antimony Co‐Contamination Influences on Soil Microbial Community Composition and Functions: Relevance to Arsenic Resistance and Carbon, Nitrogen, and Sulfur Cycling.” Environment International 153: 106522. 10.1016/j.envint.2021.106522 33812041

[mbo370426-bib-0022] Liu, G. , N. Xu , and C. Yu . 2024. “Comparative Analysis of the Microbiome of Sympatric Wintering Bean Geese, Domestic Ducks, Humans, and Soil at Shengjin Lake of China Reveals Potential Public Risk to Human Health.” Avian Research 15: 100175.

[mbo370426-bib-0023] Liu, G. , C. Yu , H. Hou , and H. Geng . 2025. “Comparative Analysis of Heavy Metals, Gut Microbial Community and Metabolome Between Bean Geese (*Anser fabalis*) Wintering at Caizi and Shengjin Lakes, China.” Global Ecology and Conservation 62: e03781.

[mbo370426-bib-0024] Liu, J. , J. Liang , X. Yuan , et al. 2015. “An Integrated Model for Assessing Heavy Metal Exposure Risk to Migratory Birds in Wetland Ecosystem: A Case Study in Dongting Lake Wetland, China.” Chemosphere 135: 14–19. 10.1016/j.chemosphere.2015.03.053 25876031

[mbo370426-bib-0025] Lozupone, C. , M. E. Lladser , D. Knights , J. Stombaugh , and R. Knight . 2011. “UniFrac: An Effective Distance Metric for Microbial Community Comparison.” ISME Journal 5, no. 2: 169–172. 10.1038/ismej.2010.133 PMC3105689 20827291

[mbo370426-bib-0026] Maqsood, Q. , N. Hussain , M. Mumtaz , M. Bilal , and H. M. N. Iqbal . 2022. “Novel Strategies and Advancement in Reducing Heavy Metals From the Contaminated Environment.” Archives of Microbiology 204, no. 8: 478. 10.1007/s00203-022-03087-2 35831495

[mbo370426-bib-0027] Meziane, M. A. , J. F. Deliège , I. Sadougui , et al. 2026. “Microbial Strategies for Heavy Metal Remediation: Mechanisms, Challenges, and Promising Approaches for Metal Detoxification.” Environmental Science and Pollution Research 33, no. 7: 2497–2516. 10.1007/s11356-026-37486-5 41665768

[mbo370426-bib-0028] Pacwa‐Płociniczak, M. , T. Płociniczak , D. Yu , et al. 2018. “Effect of Silene Vulgaris and Heavy Metal Pollution on Soil Microbial Diversity in Long‐Term Contaminated Soil.” Water, Air, and Soil Pollution 229: 13. 10.1007/s11270-017-3655-3 PMC5754377 29367788

[mbo370426-bib-0029] Proshad, R. , T. Kormoker , M. Abdullah Al , M. S. Islam , S. Khadka , and A. M. Idris . 2022. “Receptor Model‐Based Source Apportionment and Ecological Risk of Metals in Sediments of an Urban River in Bangladesh.” Journal of Hazardous Materials 423, no. Pt A: 127030. 10.1016/j.jhazmat.2021.127030 34482078

[mbo370426-bib-0030] Schloss, P. D. , S. L. Westcott , T. Ryabin , et al. 2009. “Introducing Mothur: Open‐Source, Platform‐Independent, Community‐Supported Software for Describing and Comparing Microbial Communities.” Applied and Environmental Microbiology 75, no. 23: 7537–7541. 10.1128/AEM.01541-09 PMC2786419 19801464

[mbo370426-bib-0031] Schwantes, D. , A. C. Gonçalves Junior , J. Manfrin , et al. 2021. “Distribution of Heavy Metals in Sediments and Their Bioaccumulation on Benthic Macroinvertebrates in a Tropical Brazilian Watershed.” Ecological Engineering 163: 106194.

[mbo370426-bib-0032] Segata, N. , J. Izard , L. Waldron , et al. 2011. “Metagenomic Biomarker Discovery and Explanation.” Genome Biology 12, no. 6: R60. 10.1186/gb-2011-12-6-r60 PMC3218848 21702898

[mbo370426-bib-0033] Shen, H. , and L. Guo . 2025. “Evaluation and Source Analysis of Heavy Metal Pollution in Sediments of Wetlands in Wanjiang Economic Belt.” Ningxia Journal of Agriculture and Forestry Science and Technology 66, no. 04: 10–16, 52 (in Chinese With English Abstract).

[mbo370426-bib-0034] Shi, W. , and X. Ma . 2017. “Effects of Heavy Metal Cd Pollution on Microbial Activities in Soil.” Annals of Agricultural and Environmental Medicine 24: 722–725. 10.26444/aaem/80920 29284254

[mbo370426-bib-0035] Sun, W. , E. Xiao , Y. Dong , et al. 2016. “Profiling Microbial Community in a Watershed Heavily Contaminated by an Active Antimony (Sb) Mine in Southwest China.” Science of the Total Environment 550: 297–308. 10.1016/j.scitotenv.2016.01.090 26820933

[mbo370426-bib-0036] Sun, W. , E. Xiao , T. Xiao , et al. 2017. “Response of Soil Microbial Communities to Elevated Antimony and Arsenic Contamination Indicates the Relationship Between the Innate Microbiota and Contaminant Fractions.” Environmental Science & Technology 51: 9165–9175. 10.1021/acs.est.7b00294 28700218

[mbo370426-bib-0037] Tong, Y. , G. Lin , X. Ke , et al. 2005. “Comparison of Microbial Community Between Two Shallow Freshwater Lakes in Middle Yangtze Basin, East China.” Chemosphere 60: 85–92. 10.1016/j.chemosphere.2005.01.037 15910906

[mbo370426-bib-0038] Wang, C. , B. Zhu , Y. Guo , S. Tian , Z. Zhang , and X. Hou . 2022. “Assessment of the Pollution of Soil Heavy Metal(loid)s and Its Relation With Soil Microorganisms in Wetland Soils.” Sustainability 14: 12164.

[mbo370426-bib-0039] Wang, Y. , W. Wang , Z. Zhou , W. Xia , and Y. Zhang . 2021. “Effect of Fast Restoration of Aquatic Vegetation on Phytoplankton Community After Removal of Purse Seine Culture in Huayanghe Lakes.” Science of the Total Environment 768: 144024. 10.1016/j.scitotenv.2020.144024 33736347

[mbo370426-bib-0040] Xia, J. , X. Fan , Y. Lu , et al. 2025. “Geochemical Behavior of Iron‐Sulfur Coupling in Coastal Wetland Sediments and Its Impact on Heavy Metal Speciation and Migration.” Marine Environmental Research 207: 107065. 10.1016/j.marenvres.2025.107065 40085984

[mbo370426-bib-0041] Xia, P. , L. Ma , Y. Yi , and T. Lin . 2021. “Assessment of Heavy Metal Pollution and Exposure Risk for Migratory Birds‐ A Case Study of Caohai Wetland in Guizhou Plateau (China).” Environmental Pollution 275: 116564. 10.1016/j.envpol.2021.116564 33581637

[mbo370426-bib-0042] Xiao, L. , Z. Yu , H. Liu , et al. 2020. “Effects of Cd and Pb on Diversity of Microbial Community and Enzyme Activity in Soil.” Ecotoxicology 29: 551–558. 10.1007/s10646-020-02205-4 32394358

[mbo370426-bib-0043] Yang, H. , G. G. Chen , H. Y. Liu , et al. 2013. “Pollution of Heavy Metals in the Sediments From the Lower Reaches of the Yangtze River and Its Potential Ecological Risk Assessment.” Earth and Environment 41, no. 02: 160–165 (in Chinese With English Abstract).

[mbo370426-bib-0044] Yang, L. , L. Zhou , and Y. Song . 2015. “The Effects of Food Abundance and Disturbance on Foraging Flock Patterns of the Wintering Hooded Crane (*Grus monacha*).” Avian Research 6: 15.

[mbo370426-bib-0045] Yin, D. , F. Peng , T. He , Y. Xu , and Y. Wang . 2020. “Ecological Risks of Heavy Metals as Influenced by Water‐Level Fluctuations in a Polluted Plateau Wetland, Southwest China.” Science of the Total Environment 742: 140319. 10.1016/j.scitotenv.2020.140319 32634688

[mbo370426-bib-0046] Zhang, M. , J. Zhou , F. Ge , et al. 2019. “Distribution and Contamination Evaluation of Heavy Metals in the Sediments From Shengjin Lake National Nature Reserve.” Ecological Science 38, no. 1: 9–16 (in Chinese With English Abstract).

[mbo370426-bib-0047] Zhang, W. , J. Han , H. Wu , et al. 2021. “Diversity Patterns and Drivers of Soil Microbial Communities in Urban and Suburban Park Soils of Shanghai, China.” PeerJ 9: e11231. 10.7717/peerj.11231 PMC8053383 33959419

[mbo370426-bib-0048] Zhou, Y. , M. Clark , J. Su , and C. Xiao . 2015. “Litter Decomposition and Soil Microbial Community Composition in Three Korean Pine (*Pinus koraiensis*) Forests Along an Altitudinal Gradient.” Plant and Soil 386: 171–183.

[mbo370426-bib-0049] Zhu, D. , Z. Wang , and Z. Zhang . 2023. “Effects of Heavy Metal Pollution and Soil Physicochemical Properties on the Sphagnum Farmland Soil Microbial Community Structure in Southern Guizhou, China.” International Journal of Phytoremediation 25: 1762–1773. 10.1080/15226514.2023.2191139 36949727

[mbo370426-bib-0050] Zuo, Y. , Y. Li , H. Chen , G. Ran , and X. Liu . 2023. “Effects of Multi‐Heavy Metal Composite Pollution on Microorganisms Around a Lead‐Zinc Mine in Typical Karst Areas, Southwest China.” Ecotoxicology and Environmental Safety 262: 115190. 10.1016/j.ecoenv.2023.115190 37390724

