# Supplementary material for: Heavy Metal Characteristics and Their Association With Microbial Communities of Wetland Soils of Shengjin and Caizi Lakes, China
Source: Microbiologyopen. 2026 Sep 30;15(5):e70426. doi: 10.1002/mbo3.70426 (PMC13625968; doi:10.1002/mbo3.70426)
Supplement: Supplementary file 1 [file MBO3-15-e70426-s001.docx]

**Supplementary Table**

Supplementary Table 1 Soil sampling sites and corresponding sample sizes collected from Shengjin Lake and Caizi Lake

| Lakes | Code | Sampling site | Sample size |
| --- | --- | --- | --- |
| Shengjin Lake | ZH | Zhouhe Village | 2 |
|  | JS | Jinshan Village | 2 |
|  | CA | Chi’an Village | 2 |
|  | YET | Yang’etou Village | 2 |
|  | HGXC | Hongguang Xincun Village | 2 |
| Caizi Lakes | SDGY | Wetland Park | 2 |
|  | XZT | Wangjiaban Village | 2 |
|  | TJDW | Tuanjie Dawei | 2 |
|  | CFD | Chefudao Island | 2 |
|  | XR | Xianrang Village | 2 |

Supplementary Table 2 Standard solutions for plotting the curves

| Elements | Reference number | Vendor | Detection methods | Determination limits(mg·kg^-1^) |
| --- | --- | --- | --- | --- |
| As | GBW08611 | NIM^a^ | AFS^b^ | 0.04 |
| Hg | GBW08617 | NIM | AFS | 0.01 |
| Pb | GBW08619 | NIM | ICP-MS^c^ | 0.80 |
| Cd | GBW08612 | NIM | ICP-MS | 0.03 |
| Cr | GBW08784 | NIM | ICP-MS | 1.60 |

^a^ NIM: National Institute of Metrology, Beijing, China

^b^ AFS: atomic fluorescence spectrometry

^c^ ICP-MS: inductively coupled plasma mass spectrometer

Supplementary Table 3 Measurements of the required metal concentrations of the certified reference material

| Elements | Certified values (mg∙kg^-1^) | Measured values (mg∙kg^-1^) | Relative errors |
| --- | --- | --- | --- |
| As | 15.8±0.9 | 15.4 | 2.53% |
| Hg | 0.075±0.007 | 0.071 | 5.33% |
| Pb | 40±2 | 41.5 | 3.75% |
| Cd | 0.106±0.007 | 0.104 | 1.89% |
| Cr | 62±2 | 61.2 | 1.29% |

**Supplementary Figures**

Supplementary Figure 1 Dominant microbial taxa at the phylum (A), class (B), order (C), family (D), and genus (E) levels in soil samples from Shengjin Lake (SJ) and Caizi Lake (CZ).
